# The Management of Hepatic Encephalopathy from Ward to Domiciliary Care: Current Evidence and Gray Areas

**DOI:** 10.3390/jcm13010166

**Published:** 2023-12-27

**Authors:** Daniele Bellafante, Stefania Gioia, Jessica Faccioli, Oliviero Riggio, Lorenzo Ridola, Silvia Nardelli

**Affiliations:** Department of Translational and Precision Medicine, “Sapienza” University of Rome, 00185 Rome, Italy; dan.bellafante@gmail.com (D.B.);

**Keywords:** overt hepatic encephalopathy, minimal hepatic encephalopathy, portosystemic shunts, ammonia, liver cirrhosis

## Abstract

Hepatic encephalopathy (HE) is a common complication of advanced liver disease and acute liver failure. It is a condition that features several neuropsychiatric symptoms that affect mortality, morbidity and the quality of patients’ and caregivers’ lives. An HE diagnosis is generally an exclusion diagnosis. Once the patient is admitted to the hospital, clinical examination, blood tests and eventually neuroimaging should be performed with the aim of ruling out other causes of acute brain dysfunction. Moreover, HE is recognized using various precipitants that can potentially promote its onset, alone or in combination, and must be identified. Once the diagnostic process is complete, a correct treatment should be started. The anti-HE treatment is based on a combination of the correction of precipitants; non-absorbable antibiotics, such as rifaximin; and non-absorbable disaccharides. Once the patient is discharged from the hospital, specific anti-HE therapy should be maintained in order to prevent other HE episodes.

## 1. Introduction

Hepatic encephalopathy (HE) is one of the major complications of advanced liver disease and acute liver failure [1]. HE is characterized by a spectrum of neuropsychiatric symptoms ranging from mild cognitive impairment to more severe manifestations, such as a coma [2]. The most common is type C HE, which is associated with the presence of chronic liver disease with portal hypertension or systemic shunting [3]. According to the West Haven criteria, overt HE can be classified into four grades according to the severity of symptoms [3,4]:

- Grade I: shortened attention span, lack of awareness and mood changes.
- Grade II: disoriented in space (oriented in time), flapping tremor and inappropriate behavior.
- Grade III: disoriented in time and space, somnolence but responding to verbal stimuli and confusion (Glasgow Coma Scale GCS > 8).
- Grade IV: coma (GCS < 8).

Based on the time course and frequency of relapse [2,5], overt HE can be divided into the following categories:

- Episodic: generally precipitated by one or more factors that must be identified and treated.
- Recurrent: when two or more episodes occur within 6 months.
- Persistent: when the patient does not return to their baseline mental performance between bouts of HE.

Overt hepatic encephalopathy’s (OHE’s) prevalence in patients with cirrhosis ranges between 10% and 20% [3,6]. However, the reported prevalence can vary widely in different studies and populations. It is important to note that hepatic encephalopathy can occur at any stage of cirrhosis, and its prevalence tends to increase as the severity of liver disease progresses [7]. The overall incidence of HE in patients with a transjugular intrahepatic portosystemic shunt (TIPS) ranges between 18% and 45% [3,6,7], and refractory HE post-TIPS can occur in 3–8% of patients [8].

When HE develops, mortality and worse clinical outcomes are increased in patients with liver disease [9]. It was shown that the overall survival of cirrhotic patients with HE is less than 50% at one year and less than 25% at three years [10]. A retrospective study by Ventura-Cots M. et al. demonstrated that the duration of an acute episode of HE impacts the prognosis. In their study, patients with cirrhosis and an episode of HE > 48 h had lower transplant-free survival rates compared with those with less time with HE [11]. The impact of HE on mortality in patients who have undergone TIPS implantation is uncertain, and thus, more studies are needed to assess the correlation.

Minimal hepatic encephalopathy (MHE) is a subtle and early form of hepatic encephalopathy. Unlike overt hepatic encephalopathy, where symptoms are more pronounced, individuals with MHE may not display obvious clinical signs of cognitive impairment or altered mental status, making it challenging to diagnose based on traditional clinical assessments alone [5]. MHE is characterized by subtle neurocognitive deficits, such as mild impairment in attention, psychomotor speed, and executive functions. These cognitive changes can be identified through specialized neuropsychological testing, but they may not be apparent in routine clinical examinations. Despite the lack of overt symptoms, MHE can still impact a person’s daily functioning and quality of life [12].

The exact mechanisms that lead to hepatic encephalopathy are not fully understood, but it is believed to be related to the accumulation of toxic substances, particularly ammonia, in the bloodstream [13]. The primary factor contributing to hepatic encephalopathy is the liver’s inability to metabolize and detoxify substances normally, leading to their accumulation in the body, particularly the brain. In a healthy liver, ammonia is converted into urea and excreted from the body. However, in liver disease, this process is impaired, leading to elevated levels of ammonia that can affect the brain. Ammonia is primarily generated in the gut as the product of three biochemical reactions: protein digestion, amino acid deamidation and bacterial urease activity. In addition to this, in the muscles, brain and kidneys, ammonia is produced and used in numerous biochemical reactions, such as the amination of glutamate and the deamidation of glutamine via glutamine synthetase and glutaminase [1,13]. In patients with altered liver function due to acute or chronic disease, the main detoxification pathway of ammonia is compromised, leading to high concentrations of ammonia in the blood [14]. Other than this, in patients with spontaneous portosystemic shunts or TIPS, blood from the gut skips the liver because of the blood flow diversion due to the shunts. The consequence is the transportation of higher levels of ammonia into the systemic circulation [5]. Ammonia exerts its deleterious effects on brain functions through multiple pathways, such as swelling and dysfunction of astrocytes, oxidative stress, mitochondrial dysfunction and alterations in membrane potential [1]. These abnormalities cause neuronal dysfunction and brain edema [15], which is the basis for the development of neurological symptoms. A direct correlation between levels of ammonia and the grade of HE has not been confirmed by studies [16], even if it is well acknowledged that ammonia is crucial in HE development. Recent works highlighted the role of other mechanisms involved in HE pathophysiology, such as systemic inflammation and intestinal microbiota dysbiosis [17,18]. These alterations lead to the production of high levels of pro-inflammatory cytokines that work synergistically with hyperammonemia to cross the blood–brain barrier and cause cellular swelling [16,17,18,19].

## 2. Management of Overt HE in Hospitalized Patients

**Figure 1:**
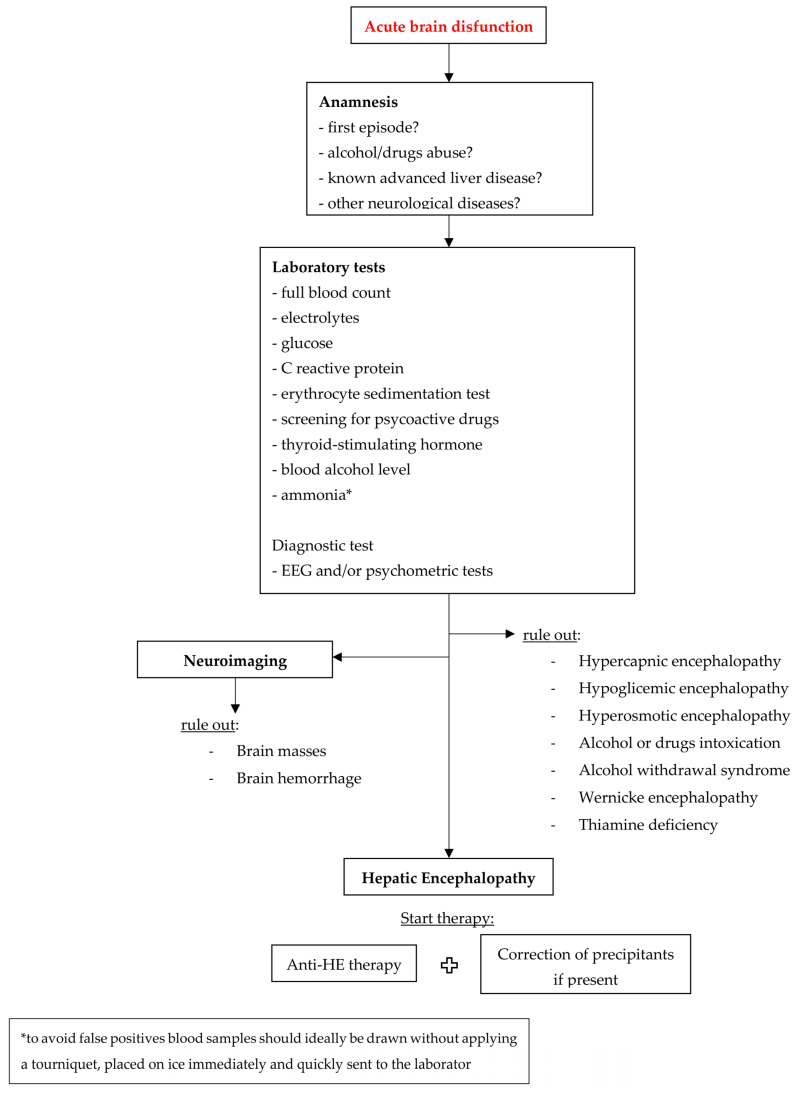
Diagnostic–therapeutic algorithm of HE.

In a hospital setting, for a patient with advanced liver disease or acute liver failure, it is important to promptly recognize HE because it affects the prognosis, leading to a worsening of clinical outcomes and increasing mortality [20]. The diagnosis of HE is not always immediate because it can overlap with other medical, neurological and psychiatric conditions that cause brain dysfunction [9]. First, an accurate anamnesis should be done, also with the precious help of family members or caregivers, when possible, with the aim to identify previous episodes of HE or a known or possible liver disease. Clinical examination and features are crucial in an HE diagnosis (flapping tremor, disorientation in time and space, changes in personality, somnolence, confusion, coma), as well as a differential diagnosis while considering other neurological disorders that may simulate HE (electrolyte alterations, hypercapnia, psychosis, dementia, meningoencephalitis, alcohol or drugs intoxication, brain masses, Wilson’s disease) [21]. Akthar AJ et al. demonstrated that 22% of patients with suspected HE and liver disease had other extrahepatic causes of acute brain dysfunction [22]. Therefore, in order to improve the prognostic accuracy and efficacy of treatments, in patients with suspected HE, it is essential to identify alternative or additional causes of neurological impairment [3]. For this reason, patients with suspected HE should undergo the same standardized diagnostic evaluation as all patients with altered consciousness [3]. As shown in Figure 1, the diagnostic work-up might include the following tests: full blood count, electrolytes, glucose, inflammatory markers (for example, C-reactive protein or erythrocyte sedimentation test), blood alcohol level, and screening for psychoactive drugs and thyroid-stimulating hormone. 

Since the level of blood ammonia plays a key role in the pathophysiology of HE, special reference needs to be made to the role of its blood determination. Measuring blood ammonia levels is a common diagnostic tool in the evaluation of patients with hepatic encephalopathy (HE). Elevated ammonia levels in the blood are associated with impaired liver function and are believed to contribute to the neurological symptoms of HE. However, it is important to note that blood ammonia levels alone do not provide a definitive diagnosis of hepatic encephalopathy, and clinical judgment, along with other diagnostic tests, is typically used to assess the severity and underlying cause of the condition. Moreover, it is important to consider that blood ammonia levels can fluctuate and may not always correlate directly with the severity of symptoms in hepatic encephalopathy. Other factors, such as dietary protein intake, medications and the presence of gastrointestinal bleeding, can influence ammonia levels. Initially, it was thought that ammonia levels should be measured using arterial blood, but studies demonstrated a similar correlation between the clinical severity of HE and arterial partial pressure of NH_3_ or ammonemia and venous ammonemia [23]. The quality of ammonemia measurements challenges the reliability of the results; in fact, to avoid a falsely elevated result, its measurement should be carefully performed as follows: blood samples should ideally be drawn without applying a tourniquet, placed on ice immediately and quickly sent to the laboratory [24]. Unfortunately, a similar protocol is not as easy to apply in routine practice as it is in research protocols [23]. Because of these difficulties, the utility of performing ammonia levels is debated and not systematically recommended [24]. But, when properly performed, ammonia levels could help in the differential diagnosis between HE and acute encephalopathy due to other causes because a normal blood ammonia level has a negative predictive value in excluding HE [23,24]. The prognostic role of ammonia remains unclear [16], but a recent multicenter study in acute-on-chronic liver failure found that plasma ammonia correlates not only with the severity of HE but also with the failure of other organs and that the lack of amelioration in ammonia levels is associated with a high risk of death [25].

A specific radiological sign of HE does not exist, but a CT scan or MRI might be performed in case of diagnostic doubts and to exclude other causes of acute brain dysfunction.

Concerning the use of neurophysiological tests in an OHE diagnosis, the electroencephalogram (EEG) is not commonly used. Nowadays, its utilization is more related to assessing minimal hepatic encephalopathy (MHE) in research fields [26]. Nevertheless, an EEG allows for identifying changes in cortical activity in uncooperative patients (a progressive slowing of general activity, an initial increase and then decrease of the wave’s amplitude, and the presence of three-phase waves, which, however, are not specific for HE). Therefore, EEG and neurophysiological investigations, in combination with a clinical examination, can find their usefulness in ruling out disturbances of consciousness due to other causes (i.e., drug-induced disturbances) and monitoring the improvement or worsening of brain function of HE in the follow-up period [27].

The management of hepatic encephalopathy (HE) in a hospital setting involves addressing the underlying liver disease, managing precipitating factors and providing supportive care to improve neurological symptoms. It is acknowledged that HE is triggered by one or more precipitating factors and then, once the diagnosis of HE is achieved, every attempt should be made to detect single or multiple precipitants and implement appropriate corrective measures [21]. One of the major causes of worsening liver function is infections [28]. They increase mortality, morbidity and the rate of other complications (ascites, gastrointestinal variceal bleeding, kidney failure) [28]. The prevalence of bacterial infections is around 25–46% in patients admitted to the hospital with decompensated cirrhosis [21]. A multicenter prospective study by Piano S. et al. tried to better define infection epidemiology in cirrhotic patients. They found that the prevalence of multidrug-resistant bacteria was 34% and that it differed significantly between geographic areas, with the greatest prevalence in Asia [29]. Another European prospective study by Fernandez et al. highlighted that infections caused by bacteria resistant to the main antibiotic families are prevalent in patients with cirrhosis, representing a growing and complex healthcare problem in patients with advanced liver disease [30]. A higher Child–Pugh score or MELD score, ascites and gastrointestinal bleeding are directly related to susceptibility to develop infections [31], and for this reason, every patient with decompensated cirrhosis admitted to the hospital should be tested for potential infections. When suspecting pneumonia, in addition to an accurate anamnesis of fever, dyspnea and other respiratory symptoms and classical blood test (CPR and leucocyte count), a chest X-ray or high-definition CT scan must be performed to identify or rule out the presence of compatible parenchymal infiltrates. If a urinary tract infection is suspected, an anamnesis for recent changes in urination and the execution of a urine test and urinoculture are necessary. If the patient has objective ascites, it is mandatory to perform a diagnostic paracentesis to exclude the presence of spontaneous bacterial peritonitis (SBP). Blood cultures must be executed if bloodstream or systemic infections are suspected. To search for acute infective gastroenteritis, an anamnesis of diarrhea with or without fever and the presence of pathogens in the stool should be performed. Gastrointestinal bleeding is associated with an increase in blood ammonia, which is part of the reason that it is another major precipitant of HE [32]. The incidence of HE after GI bleeding is 10–39% in patients with Child–Pugh B or C cirrhosis [1]. In a hospitalized patient with decompensated cirrhosis and HE, signs of recent GI bleeding (hematemesis, melena) should be investigated and an appropriate therapy must be promptly initiated. There are conflicting data on the primary prophylaxis of HE in patients with acute gastrointestinal bleeding. Different RCTs showed that the use of lactulose in cirrhotic patients hospitalized for acute gastrointestinal bleeding is effective in preventing HE compared with a placebo [33,34], while another RCT by Rattanasupar A. et al. suggested the ineffectiveness of lactulose in preventing HE in these patients [35]. These data suggest the need for more studies to assess the efficacy of primary prophylaxis of HE in patients with decompensated cirrhosis and GI bleeding. Other precipitants that should be investigated and corrected if present are dehydration, constipation, electrolyte disorders, abuse of alcohol and use of benzodiazepines (BDZs) [21]. Recent studies highlighted the role of sarcopenia (loss of muscle mass) and myosteatosis (infiltration of muscle mass by intermuscular and intramuscular fat) in precipitate HE [36,37]. It was demonstrated that sarcopenia is a strong risk factor for the development of both MHE and OHE [36]. Because of this connection between HE and malnutrition, it was suggested to perform an early assessment of nutritional status in patients with advanced liver disease, and a correct caloric intake should be given to patients [38,39]. According to the latest EASL guidelines, in non-obese patients with advanced liver disease, a daily caloric intake of 30–40 kcal/kg/day with a protein intake of 1–1.5 g/kg/day and a diet that is rich in vegetable and dairy proteins is recommended [40]. Lastly, the presence of portosystemic shunts (spontaneous or TIPS) is strongly related to HE [41]. The size, total cross-sectional area (TSA) and morphology of spontaneous portosystemic shunts (SPSSs) are associated with the development of HE; in fact, according to recent studies, it was suggested that an SPSS larger than 1 cm, a TSA > 83 mm^2^ and splenorenal shunt are more associated than others with the development of persistent or recurrent HE [42,43]. Regarding TIPSs, the incidence of HE after a TIPS placement is around 35–50% [44,45], depending on the stent diameter used [46]. A recent RCT by Wang Q. et al. compared the incidence of post-TIPS overt HE in patients that received an 8 mm covered stent implantation vs. patients that received a 10 mm covered stent implantation. They found that a TIPS with 8 mm covered stents showed a similar shunt function to a TIPS with 10 mm stents but halved the risk of spontaneous overt HE and reduced hepatic impairment [47]. Also, Schepis F. et al. demonstrated that the dilation of stents during a TIPS placement is associated with lower rates of HE [48].

According to the latest EASL guidelines, patients with grade III or IV HE should be treated in an intensive care unit (ICU). These patients might have their respiratory function altered and, consequently, they might be not able to protect their airways. For the high risk of aspiration, they need continuous monitoring in an ICU [3].

Hepatic encephalopathy poses a substantial economic burden to the healthcare system [49], which has continued to increase in recent years [50]. Inpatient hospitalization is responsible for a large portion of the healthcare burden of HE. The burden on the healthcare system arises from several factors: hospitalization, diagnostic tests, treatment costs, follow-up care and impact on productivity; in fact, patients with HE may experience a decline in cognitive function, which can impact their ability to work and perform daily activities. This can contribute to indirect costs related to lost productivity [51]. Moreover, different studies demonstrated that patients with cirrhosis and OHE have a longer length of stay compared with patients with cirrhosis and without OHE [51,52]. On the other hand, hospital readmissions play a key role in increasing the healthcare burden; in fact, HE is a primary risk factor for hospital readmissions among cirrhotic patients [53,54]. Patients’ impaired-health-related quality of life costs are added to the direct economic aspect of healthcare use, increasing the burden of HE [50]. In addition to this, HE has been associated with significant psychological burdens to caregivers [55].

## 3. Therapeutic Management

### 3.1. Acute Episode of HE

Once OHE has been identified and diagnosed, a correct treatment should be started. Treatment of HE includes not only specific therapy aimed at reducing the blood levels of ammonia but also the correction of precipitating factors [21]. In these settings, precipitants must be managed as follows:

- Infections: Empirical antibiotic therapy should be promptly started. The choice of the antibiotic is guided by the local antibiotic resistances, type and severity of infection, and local environment. When the antibiogram is available, a specific antibiotic should be used [56].
- Variceal GI bleeding: In accordance with the Baveno VII guidelines, a combination of vasoactive drugs, antibiotic prophylaxis and specific endoscopic treatment within 12 h from the presentation of bleeding should be performed. In selected cases, the TIPS placement should be considered [57].
- Non-variceal GI bleeding: a high dosage of proton pump inhibitors should be started, and an upper endoscopy must be performed within 24 h.
- Electrolyte disorders: correction with infusion therapy.
- Dehydration: use of fluid therapy and stop diuretics if used.
- Constipation: use of oral laxatives or bowel enemas.
- Malnutrition (muscle alterations): patients must follow dietary advice and consume a correct supply of nutrients [40].
- SPSS: in the case of recurrent or persistent HE, interventional radiology should be considered with the radiological retrograde shunt obliteration (balloon-occluded, plug-assisted or coil-assisted retrograde transvenous obliteration) [58,59,60].
- TIPS: consider a TIPS revision in case of persistent post-TIPS HE.

Correction of the precipitants always precedes specific anti-HE treatment, and in most cases, the resolution of an overt HE episode can be expected with just this primary intervention [3]. Every measure to control the progression of underlying liver disease should be undertaken. Simultaneously with the correction of precipitants, specific anti-HE treatment with lactulose or lactitole enemas and per os should be administered. After the first episode of HE, secondary prophylaxis to prevent further HE episodes must be promptly started when the patient is hospitalized.

### 3.2. Secondary Prophylaxis

**Figure 2:**
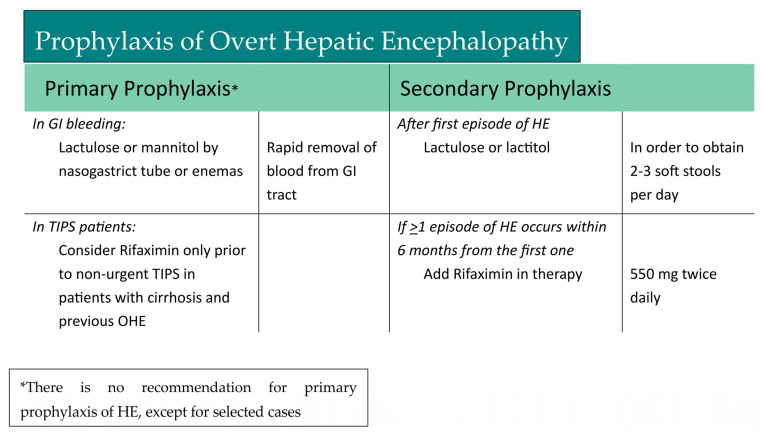
Prophylaxis of OHE.

To date, the best therapeutic strategy to prevent further episodes of HE is to maintain a low level of blood ammonia. This may involve interventions such as lactulose or lactitol; non-absorbable disaccharides that promote the excretion of ammonia in the feces; or rifaximin, which is an antibiotic that reduces the production of ammonia-producing bacteria in the gut [3]. As shown in Figure 2, after the first episode of HE, the patient should start therapy with lactulose or lactitol. Lactulose is metabolized into lactic acid and acetic acid, which increases the acidity of the intestinal environment. In this environment, ammonia (NH_3_) is converted to ammonium ions (NH_4+_), which cannot be absorbed through the intestinal barrier. Lactulose is recommended as secondary prophylaxis following a first episode of overt HE and should be titrated to obtain 2–3 bowel movements per day. If patients present with one or more new episodes of HE within six months from the first one, rifaximin should be added to the lactulose therapy [3,61,62]. Rifaximin should be administered at a dosage of 550 mg twice daily [62]. The action of rifaximin in hepatic encephalopathy is thought to be multifaceted: first, it reduces the population of ammonia-producing bacteria in the gut; moreover, rifaximin has anti-inflammatory properties that mitigate the neuroinflammation observed in hepatic encephalopathy [62]. A recent RCT by Patel VC. et al. suggested that when rifaximin exerts its action, it leads to the resolution of HE and prevents new HE episodes by reducing the likelihood of infections and attenuating systemic inflammation. Rifaximin is minimally absorbed and is thought to act locally in the gut. According to their research, rifaximin plays a role in gut barrier repair, reducing the translocation of bacteria and endotoxins from the gut to the bloodstream [63].

### 3.3. Primary Prophylaxis

To date, there is no recommendation for primary prophylaxis of HE in patients with advanced liver disease. Nevertheless, recent studies have suggested the importance of primary prophylaxis for HE in selected cases:

Gastrointestinal bleeding: As already mentioned, GI bleeding is one of the most common precipitants of HE, and data on an effective primary prophylaxis on HE in these patients are conflicting. Sharma P. et al. evaluated the role of lactulose for prophylaxis of HE after acute variceal bleeding (AVB). They compared two randomized groups of patients with AVB (lactulose vs. placebo) and they found that the majority of patients who developed HE were in the placebo group [64]. Also, in another RCT, Wen J. et al. suggested that lactulose is an effective prophylaxis agent of HE for cirrhotic patients who have developed GI bleeding [34]. Another recent RCT demonstrated that ammonia-lowering drugs (lactulose, rifaximin and L-ornithine L-aspartate) were found to be effective in preventing OHE in these patients [33]. In contrast, Rattanasupar A. et al. conducted a randomized, double-blinded, placebo-controlled, multicenter study in which they compared a lactulose group and a placebo group, showing no differences in HE incidence in the two groups, suggesting that unnecessary treatment with laxatives should be avoided in these patients [35]. These conflicting data should stimulate new research on the efficacy of pharmacological primary prophylaxis in these patients. To date, as the latest European guidelines recommend, the only effective prophylaxis is the rapid removal of blood from the gastrointestinal tract with lactulose or mannitol via a nasogastric tube or lactulose enemas [3].

TIPS: Post-TIPS HE is one of the major complications of TIPS [65]. The first trial with the purpose of discovering an effective pharmacological treatment to prevent HE after TIPS implantation was conducted by Riggio et al. in three different groups of patients submitted to TIPS treatment (placebo vs. lactitol vs. rifaximin) and they found that there was no difference in the HE incidence in the first month after the TIPS placement in the three groups [66]. More recently, in an RCT by Bureau et al., it was demonstrated that rifaximin reduced the risk for OHE in patients with cirrhosis treated with a TIPS [67]. To date, based on the few available data, the most recent guidelines stated that rifaximin can be considered for prophylaxis of HE prior to non-urgent TIPS placement in patients with cirrhosis and previous episodes of OHE [3].

## 4. Domiciliary Care

Once the patient has been dismissed from the hospital after a new episode of HE, therapy with non-absorbable disaccharides with or without a non-absorbable antibiotic should be maintained [3]. Discontinuation of therapy could be considered case-by-case only in selected patients, like those with a history of OHE, an amelioration of liver function and nutritional status, and those in whom HE was triggered by a precipitating factor that no longer exists (for example patients with HE caused by acute variceal bleeding treated with endoscopic therapy and with no more evidence of esophageal or gastric varices) [3]. More studies are needed to confirm this evidence.

In domiciliary settings, a key role in the detection of the first sign of HE in cirrhotic patients (especially in MHE, when the neuropsychological symptoms are shaded and subtle) is played by caregivers, namely, those who may notice little changes in behavior, loss of attention, delayed time of reaction, sleep disorders, falls, and a reduced ability to work and drive, and thus, communicate this information to the physicians to actuate the best strategy in order to prevent a further episode of HE.

## 5. Minimal Hepatic Encephalopathy (MHE)

MHE is associated with a low quality of life [68,69], in addition to cirrhosis progression [70] and a reduced survival rate [71]. Its incidence in cirrhotic patients ranges from 20% to 80%, and it is associated with an increased risk of developing OHE [72]. Because of these findings, in the latest EASL guidelines, it was suggested to screen patients with cirrhosis and no history of HE for MHE in the ward and/or clinic [3]. MHE could be diagnosed with psychometric and/or electrophysiological tests. These tests are based on the standard methodology, require sophisticated equipment and have a lower sensitivity than psychometric tests [72]. For this reason, in clinical practice, psychometric tests are performed more often. There is no gold standard for MHE diagnosis, which is often based on the execution of more tests, which are chosen depending on the available local norms or expertise [72]. The Animal Naming Test (ANM) is the most used neuropsychological screening test; it consists of letting the patient list the highest number of animals in 60 s [73]. A result of <15 animals is indicative of MHE. The Psychometric Hepatic Encephalopathy Score (PHES) is another useful test; it includes a battery of “paper-pencil” tests for the assessment of psychomotor speed and skill, set shifting, visuospatial orientation, attention, memory and concentration. Others are the Critical Flicker Frequency (CFR), Continuous Reaction Time Test (CRT) and Inhibitory Control Test (ICT) [72], but they are not commonly practiced in routine clinical settings.

Therapy in MHE: Even if rifaximin improves cognition, quality of life and driving capability [74], there are no RCTs to show that the treatment of MHE prevents overt HE [75]. The latest guidelines recommended starting therapy on a case-by-case basis [3,76,77]. Since MHE and OHE share the same pathophysiology, the therapeutic strategies are also supposed to be the same. In patients with MHE, treatment for HE could also be considered for the purpose of a differential diagnosis [3].

## 6. Conclusions

HE is one of the main complications of advanced liver disease and it has to be promptly recognized because it affects the prognosis and mortality. The diagnosis is based on the detection of symptoms and exclusion of other causes of acute brain dysfunction with the performance of an accurate anamnesis (with the help of caregivers), blood tests and radiological imaging when indicated. The diagnostic algorithm also provides for the identification of precipitating factors. The treatment is based on the correction of precipitant events and the use of non-absorbable disaccharides and non-absorbable antibiotics. To date, there is no indication for primary prophylaxis of HE in patients with cirrhosis, except for selected cases where it could be suggested (post-GI-bleeding HE and post-TIPS HE).

## Data Availability

Not applicable.
